# The Therapeutic Potential of Garlic-Derived Organic Polysulfides for Ischemia-Reperfusion Injury

**DOI:** 10.3390/ijms26178257

**Published:** 2025-08-26

**Authors:** Chunlei Wang, Ning Han, Caiyun Mao, Jiaxu Chen, Nana Cheng, Jieyou Zhao, Yunjia Song, Xutao Sun

**Affiliations:** School of Basic Medical Sciences, Heilongjiang University of Chinese Medicine, Harbin 150040, China; 13351911591@163.com (C.W.); 13214555122@163.com (N.H.); 17737493595@163.com (C.M.); 18645676662@163.com (J.C.); 18738982250@163.com (N.C.); zzjtcm@163.com (J.Z.)

**Keywords:** garlic-derived organic polysulfides, ischemia-reperfusion, inflammation, oxidative stress, apoptosis

## Abstract

Ischemia-reperfusion (I/R) injury refers to the exacerbation of tissue or organ damage upon the restoration of blood flow after an ischemic event. Despite its widespread clinical occurrence, therapeutic interventions for I/R injury remain limited in efficacy, presenting a significant challenge in modern medicine. Garlic, traditionally consumed as a food, has gained considerable attention for its medicinal properties. Numerous animal studies have shown that garlic-derived organic polysulfides significantly improve nerve function scores post-I/R, reduce infarct size, mitigate inflammatory responses, and inhibit cellular apoptosis. Thus, understanding the role of garlic-derived organic polysulfides in I/R injury may unveil novel therapeutic targets. This review explores the protective effects and mechanisms of garlic-derived organic polysulfides on I/R injury in various organs, including the brain, spinal cord, myocardium, lungs, liver, kidneys, and testes, highlighting their potential in advancing treatment strategies for affected patients.

## 1. Introduction

The term ischemia-reperfusion (I/R) refers to the physiological and pathological responses observed in ischemic tissues upon the restoration of blood flow. I/R represents a critical pathological mechanism underlying numerous complex human diseases, including stroke, myocardial infarction, acute kidney failure, and other conditions with significant societal implications [1]. Ischemia leads to hypoperfusion and hypoxia, which can result in cell death and tissue necrosis due to an insufficient energy supply [2]. Typically caused by acute arterial occlusion, as seen in ischemic cerebrovascular accidents, myocardial infarctions, and limb ischemia, ischemia is managed through reperfusion therapies such as thrombolytic treatment, angioplasty, surgical revascularization, and tourniquet surgery [3]. While reperfusion is crucial for preserving ischemic tissue, it may also exacerbate cellular damage by generating reactive oxygen species (ROS), triggering local inflammation, and elevating sarcoplasmic Ca^2+^ levels [4,5,6]. I/R injury can affect various organ systems, including the heart, brain, lungs, liver, kidneys, and skeletal muscles [7,8]. Characterized by a complex interplay of mechanisms, I/R injury involves mitochondrial dysfunction, impaired ion transport, electrolyte imbalances, altered enzymatic activity, increased cellular water influx, and resultant cell swelling [9]. Reperfusion amplifies these issues by promoting ROS accumulation, endothelial dysfunction, inflammation, cytokine release, cellular damage, and apoptosis [10]. Inflammation plays a central role in the pathogenesis of I/R injury [11]. Despite progress in medical research, I/R injuries remain a significant clinical challenge, emphasizing the need for improved therapeutic strategies.

Historically, *Allium sativum* (garlic) has been used as a medicinal remedy for a variety of ailments, including diarrhea, infections, asthma, and leprosy, throughout ancient, medieval, and modern times [12,13]. Contemporary pharmacological studies have confirmed the anti-inflammatory, antioxidant, immunomodulatory, and anti-catabolic effects of garlic’s active compounds [14,15,16,17,18]. The main active ingredients in garlic include allicin, diallyl sulfide (DAS), diallyl disulfide (DADS), diallyl trisulfides (DATS), s-allyl-cysteine (SAC), and s-allyl-l-cysteine (SAMC) [15]. Allicin, one of garlic’s most potent constituents, has been shown to be effective in treating I/R injury, as demonstrated by Zhao et al.’s research [19,20]. The cost-effectiveness and widespread availability of garlic, combined with its significant therapeutic potential, have contributed to its popularity as an alternative treatment for I/R injury.

This review explores the effects of garlic-derived organic polysulfides on I/R injury in various tissues and organs, focusing on the regulatory mechanisms of allicin and recent advancements in I/R treatment. It aims to facilitate the development of new garlic-based therapeutic agents and offer alternative clinical strategies for managing I/R injuries.

## 2. Sources of Garlic and Its Active Substances

The phytochemical composition of garlic is characterized by a diverse range of biologically active compounds, including organosulfur derivatives, saponin-based molecules, phenolic acids, and polysaccharide polymers [21]. Garlic contains 60–65% water, 28–30% carbohydrates, approximately 2.3% organic sulfur compounds, 2–6% protein, approximately 1.2% amino acids, and approximately 1.5% fiber, fatty acids, and trace mineral elements [22,23]. Damage to garlic bulbs triggers the enzymatic conversion of thiocysteine sulfoxide into thiosulfinate, which, under the action of alliinase, produces allicin. Allicin, a thiosulfinate, was first structurally identified by Stoll and Seebeck in 1948 [24]. The precursor to allicin is the non-protein amino acid alliin (SAMC) [25]. However, studies show that allicin decomposes at 20 °C over 20 h into DADS (66%), DAS (14%), DATS (9%), and other compounds [26]. These derivatives also exhibit significant biological activities, including antioxidant, antibacterial, and antiviral properties. Additionally, garlic is rich in both water-soluble and fat-soluble organic sulfides, such as SAC and SAMC, which also have notable pharmacological effects (Table 1).

## 3. Prevention and Treatment of I/R in Different Tissues by Garlic Derivatives

### 3.1. Impact of Garlic Derivatives on Central Nervous System I/R

#### 3.1.1. Impact of Garlic Derivatives on Cerebral I/R

The brain is highly susceptible to ischemia and hypoxia, with ischemic stroke, resulting from cerebral artery occlusion, being the second most common cause of disability worldwide [56]. Despite advancements in stroke imaging and treatment, the severe consequences of brain I/R injury, both individually and societally, remain challenging to address [57]. Understanding the molecular mechanisms underlying brain I/R and developing more effective therapies is critical. A variety of drugs, especially those derived from plants, show dual-phase therapeutic efficacy, addressing both initial hypoxic-ischemic damage and preventing subsequent reperfusion complications [58]. Furthermore, studies indicate that garlic extract is effective in treating brain I/R. Notably, allicin pretreatment significantly improves nerve function recovery in global brain I/R and reduces cerebral infarction volume [59].

**Figure 1:**
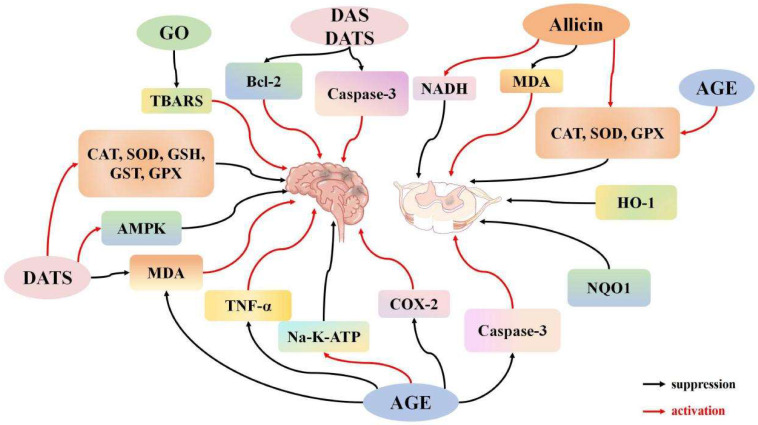
The regulatory effect of garlic derivatives on I/R of the central nervous system.

Brain I/R injury is driven by various pathogenic factors, including oxidative stress, inflammation, and apoptosis [60,61,62,63]. Redox imbalance plays a significant role in the onset of I/R injury [64]. Mitochondrial dysfunction during ischemia triggers oxidative stress, which intensifies upon reperfusion and leads to cell death via direct damage to macromolecules [65]. Notably, ROS act as dual-function signaling molecules in this process. Low levels of local ROS are crucial for maintaining intracellular homeostasis through pathways such as MAPK/ERK and PI3K-AKT. However, excessive ROS not only induces gene mutations but also causes irreversible oxidative modifications to proteins, lipids, and carbohydrates, impairing their functions and promoting disease or cell death [66]. Garlic enhances the activity of superoxide dismutase (SOD) and glutathione (GSH) antioxidant enzymes, while reducing the levels of the inflammatory factor TNF-α, thus alleviating oxidative stress and inflammation-induced tissue damage [67]. Gupta et al. [34] established a brain I/R model by blocking the right and left common carotid arteries of Swiss albino mice for 10 min, followed by reperfusion for 24 h. They found that garlic oil significantly reduced cerebral infarction size and motor incoordination induced by brain I/R. Additionally, garlic oil downregulated mitochondrial thiobarbituric acid-reactive substances (TBARS), which are markers of oxidative stress, suggesting that garlic oil mitigates ROS-induced oxidative stress and reduces brain I/R damage. Malondialdehyde (MDA), a byproduct of lipid oxidative breakdown, serves as an indirect measure of ROS levels. Sun et al. [37] anesthetized rats with chloral hydrate (350 mg/kg, intraperitoneal injection) and performed mechanical ventilation. The left subclavian artery was exposed through a left fourth intercostal incision, and after systemic heparinization (200 IU/kg), the aorta was clamped to induce ischemia, while maintaining femoral artery blood pressure at 10 mmHg to ensure ischemia. Following 14 min of ischemia, reperfusion was initiated. DATS was shown to reverse the increase in ROS levels, downregulate MDA levels, enhance the activity of SOD, GSH, and glutathione peroxidase (GPX), and mitigate nerve function damage. Prolonged ischemia, without a corresponding change in reperfusion time, induces inflammation and oxidative stress. Colín-González et al. [51] demonstrated that TNF-α levels and cyclooxygenase-2 (COX-2) protein expression increased after 60 min of ischemia followed by 24 h of reperfusion. Aged garlic extract (AGE) significantly reduced TNF-α levels, alleviated neurological changes by 61.6%, reduced infarct area by 54.8%, and decreased histological lesions by 37.7%. AGE also modulated COX-2 expression and activity, reducing COX-2-mediated inflammation and neural damage. Saleem et al. [36] induced cerebral I/R by blocking the rat mesencephalic artery for 2 h, followed by reperfusion for 22 h. Following AGE pretreatment, they observed increased levels of GSH, catalase (CAT), and SOD, alongside decreased MDA expression. Although GPX and GST activities notably declined after I/R, AGE mitigated this reduction. AGE also restored Na^+^-K^+^-ATPase activity, suggesting that it markedly reduced brain I/R-induced tissue injury by modulating COX-2 expression and enhancing antioxidant and anti-inflammatory responses. Cell apoptosis is regulated by two primary pathways: the endogenous (mitochondria-mediated) and exogenous (receptor-mediated) pathways. In the endogenous pathway, Bcl-2 antagonizes Bax, counteracting its pro-apoptotic effects through direct interaction [68]. Bcl-2 prevents apoptosis by inhibiting Bax release and oligomerization [69,70]. Furthermore, as an anti-apoptotic protein, Bcl-2 reduces lipid peroxidation, prevents the outflow of Ca^2+^ from the endoplasmic reticulum, and lowers free radical levels [71]. These effects contribute to maintaining mitochondrial function and inhibiting endogenous apoptosis. Allicin reduces neuronal apoptosis by inhibiting caspase-3 activation, increasing Bcl-2 levels, and decreasing Bax expression [72,73]. Lin et al. [45] developed a cerebral I/R model by occluding the rat middle cerebral artery for 2 h, followed by 24 h of reperfusion, with DAS administered prior to the occlusion. The results demonstrated that DAS pretreatment effectively reduced cerebellar infarction volume. DAS pretreatment inhibited the expression of activated caspase-3, enhanced Bcl-2 expression, and increased the number of Bcl-2-positive cells. These findings suggest that DAS preconditioning may protect the brain from I/R injury by modulating apoptosis. Additionally, Sun et al. [37] showed that DATS reversed the decrease in Bcl-2 levels and the increase in Bax and cleaved caspase-3 expression. DATS also activated AMPK, increasing its phosphorylation, which alleviated mitochondrial damage and reduced ROS levels. As an antioxidant, DATS works by activating AMPK, maintaining mitochondrial homeostasis, and mitigating oxidative stress-induced neuronal damage. These results collectively indicate that garlic and its polysulfide derivatives have protective effects against brain I/R injury. However, the literature does not address whether garlic and its derivatives can cross the blood–brain barrier (BBB) or provide pharmacokinetic data. It was found that polysulfides with similar structures, such as dimethyl trisulfide (DMTS), have been shown to penetrate the BBB through in vitro models and animal studies [74]. Currently, no studies exist on the ability of garlic-derived polysulfides to cross the BBB, highlighting a limitation in the existing research, and providing a direction for future experimental verification of garlic-derived polysulfides’ ability to penetrate the BBB (Figure 1).

#### 3.1.2. Effects of Garlic Derivatives on Spinal Cord I/R

The spinal cord, a crucial component of the central nervous system, is particularly vulnerable to I/R injury following trauma, degeneration, tumor compression, or aortic surgery. Spinal cord I/R injury can result in significant impairment of both sensory and motor functions [75,76,77]. Zhu et al. [40] established a rabbit spinal cord I/R model, administering allicin at various concentrations (1, 10, 50 mg/kg) for two weeks prior to the procedure. The results indicated that allicin reduced infarct size in a dose-dependent manner, significantly improved neurological dysfunction caused by spinal cord I/R, and enhanced the activities of CAT, SOD, and GPX. These enzymes protect spinal cord tissue from oxidative stress by mitigating ROS-induced damage and activating downstream metabolic enzymes, such as heme oxygenase-1 (HO-1) and NAD(P)H quinone oxidoreductase-1 (NQO1), thereby establishing an additional defense mechanism [78,79]. HO-1 is the rate-limiting enzyme in heme degradation. This process generates biliverdin, which can be rapidly converted to bilirubin by biliverdin reductase. Bilirubin, a potent antioxidant, directly eliminates ROS. Another product, carbon monoxide (CO), indirectly inhibits ROS production by temporarily inhibiting mitochondrial complex IV at low concentrations [80]. Although the detailed regulatory mechanisms of HO-1 on biliverdin and CO remain incompletely understood, existing evidence clearly supports the role of these products as key mediators of HO-1’s protective effect in I/R injury. The protective mechanism of HO-1 involves a synergistic action of these pathways. Furthermore, different concentrations of allicin were found to significantly reduce MDA levels, with percent decreases relative to controls of 319 ± 28.3%, 245 ± 41.0%, and 206 ± 35.6%, respectively. Allicin thus protects against spinal cord I/R by reducing ROS production, enhancing mitochondrial NADH levels, and inhibiting cytochrome C release, suggesting its role in improving mitochondrial function. Additionally, Cemil et al. [46] developed a spinal cord I/R model and found that AGE can reduce edema. AGE therapy significantly decreased spinal cord levels of MDA, nitric oxide (NO), and TNF-α, while increasing the activities of SOD, GSH, GPX, and CAT. Moreover, AGE reduced caspase-3 activity, thereby alleviating spinal cord neuronal damage. The study indicates that 24 h post-ischemic injury, AGE may offer neuroprotection in spinal cord I/R injury by reducing oxidative stress, lowering inflammatory cytokine levels, and inhibiting apoptosis (Figure 1).

#### 3.1.3. Effects of Garlic Derivatives on Eye I/R

Ocular I/R is a common pathological process in various blinding eye diseases, and its mechanism is closely linked to oxidative stress. The role of garlic and its derivatives in mitigating I/R-induced oxidative stress offers a theoretical foundation for their potential intervention in ocular damage. Tu et al. [81] used an H_2_O_2_-induced retinal pigmented epithelial cells (ARPE-19) model, which simulates the core characteristic of I/R injury, an increase in ROS. Allicin significantly alleviates oxidative damage by activating the Nrf2 pathway, a mechanism that aligns with the protective effects observed by He et al. [82] in a retinal I/R model. Both interventions upregulate HO-1 expression through the Nrf2/HO-1 pathway, reducing cell apoptosis. Additionally, allicin inhibits NOX4 and activates SOD, demonstrating strong antioxidant capacity, providing a theoretical basis for the expanded use of allicin in treating ischemic eye diseases.

### 3.2. Effects of Garlic Derivatives on Myocardial I/R

Ischemic heart disease remains a global health challenge, with high morbidity and mortality rates despite various treatment options [83,84]. Restoring blood flow to the ischemic myocardium enhances myocardial survival and reduces infarct size [85]. However, reperfusion often induces arrhythmias, which worsen myocardial ischemia by increasing cell necrosis, impairing cardiac function, and altering ventricular architecture [86]. Myocardial I/R results in apoptosis, necrosis, infarction, and heart failure, presenting a significant challenge in interventional cardiology [87,88,89]. Garlic has been shown to exert cardioprotective effects. Ischemic preconditioning with garlic extract, as well as garlic-extract-mediated reperfusion, significantly increases heart rate and coronary flow [43]. Early research by Bhatti et al. [27] demonstrated that garlic extract substantially reduces myocardial infarction size. Administering garlic extract during ischemic preconditioning further decreases the release of lactate dehydrogenase (LDH) and creatine kinase (CK), aiding in the treatment of I/R-induced heart damage and improving cardiac function.

#### 3.2.1. Effect of Garlic Derivatives on Cardiomyocyte Apoptosis

**Figure 2:**
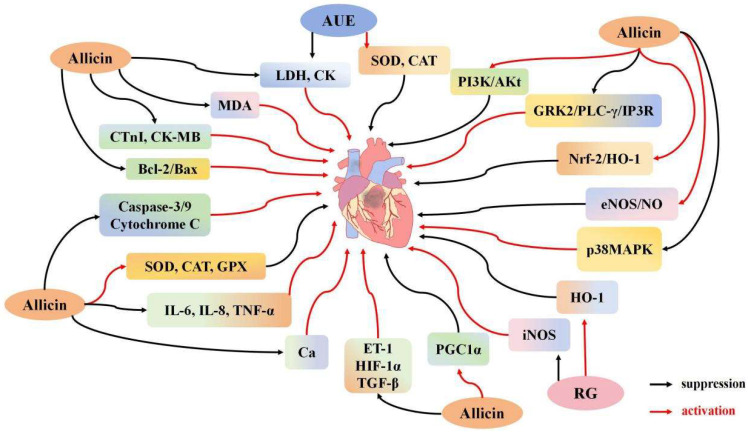
The regulatory mechanism of garlic derivatives on myocardial I/R.

Accumulating evidence suggests that myocardial I/R injury is a complex pathophysiological process involving myocardial cell apoptosis, intense inflammation, and metabolic dysfunction [90,91,92]. Ma et al. [41] demonstrated that allicin effectively improves left ventricular systolic diameter (LVID), left ventricular ejection fraction (LVEF), and stroke volume in rats, while reducing myocardial infarction size and alleviating heart damage. Allicin also lowers the levels of CK and LDH, decreases the myocardial apoptosis index, and reduces Bax expression while increasing Bcl-2 expression, with these effects being dose-dependent. These findings suggest that allicin enhances cardiac function, potentially by modulating the Bcl-2/Bax pathway and reducing cardiac cell death. In another study, Ma et al. [44] developed an ischemia/hypoxia (I/H) model that induces apoptosis in H9C2 cells. Allicin was found to decrease Ca^2+^ concentrations and improve cell morphology and viability, significantly inhibiting apoptosis by downregulating Bax and upregulating Bcl-2 expression. Deng et al. [47] established an in vitro cardiomyocyte hypoxia-reoxygenation model, demonstrating that allicin therapy enhances cellular survival by reducing apoptosis. This was marked by lower levels of Bax, caspase-3, and cytochrome C, alongside increased Bcl-2 expression. Allicin also reduced IL-6 and TNF-α levels, mitigated mitochondrial membrane potential loss, and substantially decreased intracellular ROS production. Additionally, allicin upregulated PPARγ-coactivator-1α and endothelial nitric oxide synthase (eNOS), while downregulating endothelin-1 (ET-1), hypoxia-inducible factor 1α (HIF-1α), and transforming growth factor β (TGF-β). These findings suggest that allicin protects heart cells from ischemic damage by reducing apoptosis, inflammation, and mitochondrial dysfunction. Gao et al. [42] developed an I/R model in mice by temporarily occluding the left coronary artery for 30 min, followed by 3 h of reperfusion. Allicin treatment improved LVEF and alleviated pathological changes in the left ventricular anterior wall (LVAW), enhancing cardiac function post-I/R injury. In contrast to the control group, allicin treatment reduced the size of myocardial infarctions and decreased the expression of pro-apoptotic proteins Bax and cleaved caspase-3/9. Conversely, it increased the expression of the anti-apoptotic protein Bcl-2. Furthermore, allicin influenced the PI3K/Akt signaling pathway by significantly enhancing the expression of p-Akt and p-PI3K proteins. Allicin pretreatment inhibited the upregulation of p-GRK2, p-PLC-γ, p-CaMKII, and p-IP3R, thus preventing Ca^2+^ release. These findings suggest that allicin preconditioning mitigates myocardial infarction size and apoptosis by inhibiting the PI3K-induced GRK2/PLC-γ/IP3R signaling pathway activation during I/R injury. This process helps reduce Ca^2+^ overload and mitochondrial dysfunction, thereby providing a protective effect (Figure 2).

#### 3.2.2. Effects of Garlic Derivatives on Cardiomyocyte Inflammation and Oxidative Stress

Inflammation and oxidative stress are critical factors in myocardial I/R injury [93,94]. Rankovic et al. [31] treated rats with various concentrations (125, 250, 500 mg/kg) of *Allium ursinum L.* (AUE) to address myocardial I/R. Phytochemical studies indicate that Allium ursinum L. is rich in sulfur-containing compounds (such as garlic-like substances and cysteine derivatives), flavonoids, and polyphenols. These sulfur compounds share structural similarities with well-known Allium sativum components like allicin and DADS, which are recognized for their antioxidant, anti-inflammatory, and cardioprotective effects [95]. The study found that AUE treatment led to a dose-dependent improvement in key cardiac parameters, including peak left ventricular pressure development (dp/dt max), left ventricular systolic pressure (LVSP), coronary circulation (CF), and pulse rate. The highest dose of AUE yielded the maximum SOD activity, while the lowest dose notably increased CAT activity. At all doses, AUE reversed the increase in blood oxygen markers and TBARS caused by I/R. These results suggest that higher AUE doses have a more significant impact on antioxidant enzyme activities and cardiac function recovery, demonstrating a protective role for the heart. Khatua et al. [96] observed that treatment with garlic and its metabolites reduced LDH and serum glutamate oxaloacetate transaminase (SGOT) levels, decreasing cardiac injury while upregulating NO and hydrogen sulfide (H_2_S) levels. Notably, upregulated H_2_S may exert protective effects by activating the Nrf2 signaling pathway. H_2_S promotes the nuclear localization of Nrf2, a key transcription factor that binds to antioxidant-responsive elements in the promoter regions of target genes, thereby enhancing the expression of downstream antioxidant enzymes such as GSH, SOD, and CAT [97]. Additionally, garlic and its metabolites can also increase the expression levels of GSH, SOD, and CAT, further mitigating oxidative stress-induced damage. An H9C2 cell experiment demonstrated that garlic and its metabolites reduced cell size, lowered intracellular calcium concentrations, and elevated Na^+^-K^+^-ATPase levels. However, the anti-myocardial hypertrophy effects of garlic and its metabolites were diminished in the presence of Na^+^-K^+^-ATPase inhibitors. Peng et al. [50] developed an I/R model by occluding the distal segment of the left anterior descending coronary artery for 2 h, followed by 3 h of reperfusion. The study found that allicin significantly increased LVSP and decreased left ventricular end-diastolic pressure (LVEDP) post-reperfusion, while reducing inflammatory markers such as IL-6, TNF-α, and ET-1 compared to the control group. These results suggest that allicin not only inhibits the inflammatory response during I/R but also mitigates heart damage caused by it. Liu et al. [53] induced myocardial I/R injury in male SD rats through 30 min of left coronary artery occlusion, followed by 4 h of reperfusion. After allicin treatment, LVEDP decreased, while LVSP and ±dp/dtmax absolute values increased. Allicin reduced the expression of cardiac troponin I (cTnI) and CK-MB, lowered MDA levels, and enhanced SOD, CAT, and GPX activity in serum. Furthermore, allicin decreased the expression of inflammatory cytokines, including TNF-α, IL-6, and IL-8. The study suggests that allicin protects the heart by inhibiting the p38MAPK pathway, reducing p-p38 expression, and alleviating cardiomyocyte inflammation and oxidative stress. Different concentrations of garlic homogenate reduce myocardial I/R-induced oxidative stress to varying degrees. In the 125 mg/kg and 250 mg/kg treatment groups, SOD activity significantly increased, and TBARS values decreased markedly compared to controls. In groups treated with doses over 500 mg/kg, SOD activity did not show substantial increases. Notably, the group treated with 250 mg/kg of garlic homogenate exhibited the most significant improvement in myocardial CAT activity and GSH levels. These findings indicate that 250 mg/kg of garlic homogenate provides the most effective protective effect [29]. As purification technology advances, the purity of garlic water extract has steadily increased. However, reports indicate that when the proportion of garlic water extract in feed exceeds 0.05%, it can become cardiotoxic, leading to arrhythmia and cardiac depression [43]. Furthermore, differences exist between the active ingredients of raw garlic (RG) and aged garlic (ABG). Czompa et al. [28] developed an I/R model using isolated hearts subjected to 30 min of ischemia followed by 120 min of reperfusion. Both RG and ABG exhibited significant and similar cardioprotective effects. In the model group, RG and ABG substantially reduced infarct volume. The infarct volume decreased from 27.5% to 8.4%, and after RG treatment, it further reduced to a range of 5.9% to 2.0%, while after ABG treatment, it decreased to 6.2% to 1.2%. Both RG and ABG were shown to significantly reduce myocardial infarction size and mitigate heart damage. However, the study revealed significantly lower levels of inducible nitric oxide synthase (iNOS) protein in RG-treated hearts, while ABG helped prevent the reduction of iNOS levels after I/R. Interestingly, compared to ischemic preconditioning, reperfusion with garlic extract after ischemic preconditioning substantially decreased the release of LDH following ischemia and significantly increased the release of nitrite and adenosine [43]. Additionally, allicin activates the eNOS/NO pathway, enhancing the expression of endothelial eNOS and NO, thereby promoting vasodilation and reducing inflammation and thrombotic events during I/R [44]. Furthermore, both RG and ABG treatment groups showed a marked increase in HO-1 levels in the I/R model [43]. Ma et al. found that allicin activates the Nrf2/HO-1 signaling pathway, enhancing Nrf2 expression and consequently increasing HO-1 levels [44] (Figure 2).

### 3.3. Effects of Garlic Derivatives on Lung I/R

**Figure 3:**
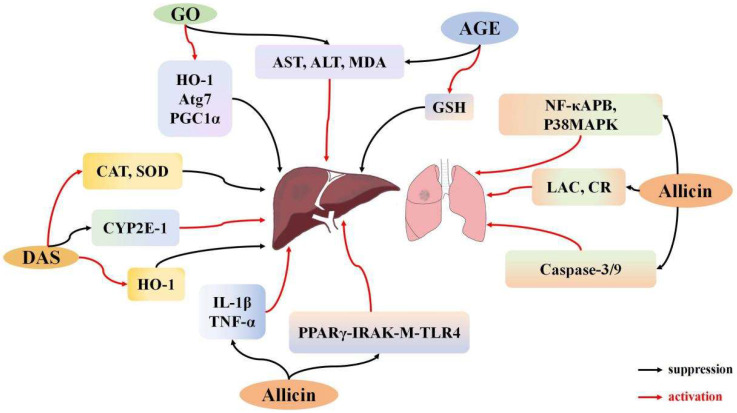
The regulatory mechanism of garlic derivatives on I/R in the lungs and liver.

Acute lung injury resulting from I/R is a complex condition marked by abnormal physiological processes that occur due to the disruption and subsequent restoration of blood supply to the lungs. The pathogenesis involves excessive production of ROS, elevated pro-inflammatory factors, and activation of the NF-κB pathway [98]. Additionally, several studies suggest that allicin can mitigate lung damage, potentially through inhibition of the p38 pathway [99]. In an early study, Batirel et al. [100] established a lung I/R model by sequentially occluding the left pulmonary artery for 1 h and the right for 2 h. Allicin was administered at the onset of reperfusion, and pulmonary artery pressure (PAP) and flow (PAF) were monitored. Allicin treatment resulted in significant reductions in both PAP and PAF, indicating its efficacy in mitigating pulmonary I/R damage. Zhang et al. [48] later built a hemorrhagic shock model of lung trauma in rats. In this model, the lower lobe of the left lung was resected, followed by 30 min of oxygen deprivation and 20 min of restoration. Allicin administration reduced levels of nuclear factor NF-κB and phosphorylated p38 MAPK. Additionally, lactate and creatinine levels were reduced, along with decreased activity and expression of caspase-3 and caspase-9. These results indicate that allicin alleviates lung cell apoptosis and lung injury through the p38 MAPK signaling pathway (Figure 3).

### 3.4. Effects of Garlic Derivatives on Liver I/R

Hepatic I/R involves the accumulation of cellular and organelle damage over time during blood flow restriction (ischemia), with further damage occurring upon blood flow restoration (reperfusion) [101]. Liver damage primarily occurs during reperfusion rather than ischemia [102]. Hepatic I/R is a common condition in various liver-related clinical scenarios and can result in liver dysfunction and failure [103,104]. During hepatic I/R, multiple pathological events, including inflammation, oxidative stress, calcium overload, and others, occur simultaneously [105,106].

Allicin protects against liver injury by reducing inflammation, oxidative stress, and cell apoptosis [107,108]. Sener et al. [38] established a liver I/R model by inducing 45 min of ischemia followed by 60 min of reperfusion in rats. Administration of AGE led to a reduction in aspartate aminotransferase (AST), alanine aminotransferase (ALT), and MDA levels. AGE also notably restored GSH levels that were diminished by I/R, highlighting its antioxidant capabilities and its role in reducing liver injury induced by I/R. Lasheen et al. [32] developed another liver I/R model in female Wistar rats with a 45-min ischemic period followed by 24 h of reperfusion. Liver I/R elevated ALT and AST levels, which were significantly lowered by garlic oil pretreatment. Serum creatinine and MDA levels were substantially reduced, while mitochondrial NAD^+^ levels increased markedly in the garlic oil pretreatment group compared to the liver I/R group. Additionally, garlic oil enhanced the expression of HO-1, Atg7, and peroxisome proliferator-activated receptor γ coactivator 1α (PGC1α) genes, further supporting the protective effects of garlic oil in mitigating liver injury. Li et al. [54] developed a liver I/R injury model by occluding the blood supply to the left and middle liver lobes, followed by intragastric administration of allicin. The study revealed that allicin suppressed transaminase activity and reduced IL-1β and TNF-α levels. A subsequent experiment explored the molecular mechanisms by using mice with IRAK-M gene knockouts and peroxisome proliferator-activated receptor-gamma (PPAR-γ) inhibitors. PPARγ inhibitors neutralized the protective effects of allicin by inhibiting PPARγ expression and also reduced IRAK-M expression. Notably, IRAK-M deficiency had no effect on PPARγ levels, emphasizing PPARγ’s critical role in allicin’s protective effect. These findings suggest that allicin mitigates liver I/R injury by modulating the PPARγ, IRAK-M, and TLR4 signaling pathways. Allicin pretreatment significantly reduces liver cell apoptosis, IL-1β and TNF-α production, and oxidative stress, offering protection through modulation of these pathways. DAS, a breakdown product of allicin, has also been shown to mitigate liver I/R injury through its antioxidant properties. Shaik et al. [35] established an I/R model in rats and found that DAS administration moderately increased NO concentration in plasma, contributing to blood vessel dilation. DAS also reduced inflammation and the generation of free radicals. Concurrently, DAS boosted CAT and SOD concentrations and markedly enhanced HO-1 expression. Furthermore, DAS treatment reduced the level of CYP2E1 protein in I/R-pretreated rats. These findings suggest that DAS mitigates liver damage by upregulating HO-1 expression and downregulating CYP2E1 expression, thereby alleviating oxidative stress-induced liver tissue damage (Figure 3).

### 3.5. Impact of Garlic Derivatives on Urinary System I/R

#### 3.5.1. Effects of Garlic Derivatives on Renal I/R

Acute kidney injury (AKI) is frequently caused by renal I/R and is characterized by a sudden decline in renal function, including decreased glomerular filtration and renal tubular reabsorption capacity. This syndrome also involves organ dysfunction, such as abnormalities in endocrine and metabolic regulation, including reduced erythropoietin secretion and disturbances in the renin-angiotensin system. With a high mortality rate, AKI may progress to chronic kidney disease [109]. Renal I/R often occurs following hemorrhagic shock, large-scale cardiovascular surgery, or kidney transplantation, leading to kidney function impairment and other complications, during which a potent inflammatory response is induced, and the kidney’s oxidative capacity is compromised [110]. Although reperfusion is critical for the survival of ischemic tissue, evidence shows that the reperfusion process itself can result in additional cellular damage [111].

**Figure 4:**
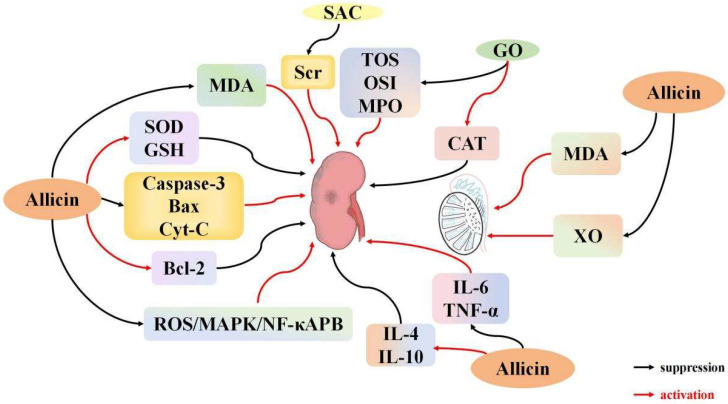
The regulatory mechanism of garlic derivatives on the urinary system I/R.

ROS generated by normal cellular metabolism are typically cleared by endogenous antioxidant enzymes, such as SOD [112]. SOD catalyzes the conversion of superoxide radicals to hydrogen peroxide (H_2_O_2_). However, excessive ROS production combined with reduced antioxidant enzyme activity exacerbates renal I/R injury [113]. Targeted interventions can reduce I/R damage by preventing mitochondrial overproduction of ROS [114,115]. Segoviano et al. [116] developed a mouse renal I/R model to investigate the protective effects of the garlic-derived antioxidant s-allyl cysteine (SAC) against renal I/R-induced injury and oxidative stress. The results revealed an increase in blood urea nitrogen levels and serum creatinine in the experimental group, yet the structural damage to the kidneys was reduced, and the intensity of 4-HNE immunostaining was significantly diminished. These findings suggest that SAC’s protective effect in renal I/R injury may be attributed to its antioxidant properties. Li et al. [49] developed a renal I/R model by inducing 45 min of ischemia in the right kidney, followed by 12 h of reperfusion. Allicin, administered via intraperitoneal injection, reduced MDA levels and mitigated the decline in SOD activity following renal I/R. Allicin also decreased the expression of caspase-3 and Bax while enhancing Bcl-2 expression. In vitro studies demonstrated that allicin attenuated apoptosis triggered by hypoxia/reoxygenation (H/R) in NRK-52E kidney cells. It appears that allicin achieved this protective effect by reducing caspase-3 and Bax production, while simultaneously increasing Bcl-2 expression. These results highlight allicin’ s antioxidant and anti-apoptotic effects, supporting its potential in promoting the repair of kidney I/R injury. Xu et al. [39] demonstrated that allicin enhances SOD and GSH levels while reducing MDA, ROS, and BUN expression by modulating the ROS/MAPK/NF-κB pathway. This study indicates that allicin protects the kidneys by reducing oxidative stress and lipid damage. Savas et al. [30] developed an I/R model by inducing bilateral renal ischemia for 45 min, followed by 6 h of reperfusion in male rats. Following I/R treatment, serum urea and creatinine levels increased, along with a decline in other kidney functions. Oral administration of garlic oil (GO) reduced the levels of serum urea and creatinine. Additionally, GO treatment led to increased CAT levels compared to the I/R group, while decreasing tissue levels of total oxidant status (TOS), oxidative stress index (OSI), and myeloperoxidase (MPO). The GO group also exhibited less pathological damage than the I/R group, suggesting that oral GO provides significant protection against renal I/R injury. Shan et al. [55] developed a renal I/R model by occluding the renal arteries and veins for 45 min, followed by 22 h of reperfusion. Allicin pretreatment enhanced antioxidant capacity by increasing SOD activity and reducing MDA levels. Allicin also elevated IL-4 and IL-10 expression while significantly reducing IL-6 and TNF-α expression. In terms of apoptosis, allicin notably reduced Bax, caspase-3, and Cyt-C expression while enhancing Bcl-2 expression, indicating its anti-apoptotic effects. These findings suggest that allicin mitigates I/R-induced kidney injury by modulating apoptosis, reducing oxidative stress, and decreasing inflammation. Combining allicin with other agents can improve its protective effects. Ali et al. [117] established a renal I/R model by occluding the left renal artery for 45 min, followed by 24 h of reperfusion. In this study, allicin combined with telmisartan was used to treat obese renal I/R. Pre-ischemia administration of either telmisartan or allicin significantly reduced serum creatinine and urea levels. Furthermore, the combination of allicin and telmisartan restored creatinine, MDA, and GSH levels to normal. This combination also effectively reduced hypercholesterolemia and hypertriglyceridemia, while decreasing adipocyte size surrounding the ischemic kidney in the obese I/R group. Telmisartan or allicin pretreatment notably reduced NF-κB immunoreactivity and TNF-α levels in ischemic renal tissue, while enhancing IL-10 expression and upregulating adiponectin receptor-1 and macrophage polarization markers (CD11c, CD206) mRNA. The combined treatment substantially inhibited the increase in these parameters, further demonstrating the efficacy of allicin in protecting against I/R injury. The protective effects of allicin were enhanced when used in conjunction with telmisartan (Figure 4).

#### 3.5.2. Effects of Garlic Derivatives on Testicular I/R

When the kidney undergoes I/R, it triggers a systemic inflammatory response and generates substantial ROS, which affects other organs through the blood circulation, including the testes. Recent research suggests potential protective effects of allicin against I/R-induced testicular damage. Unsal et al. [118] induced testicular torsion by twisting the left testicle 720° and maintaining the torsion for 2 h, followed by a 5-day pretreatment with garlic extract in rats. The results showed that garlic extract pretreatment prevented the increase in MDA and xanthine oxidase (XO) levels. Additionally, the garlic extract pretreatment reduced testicular torsion and induced the separation of germinating cells in the spermatotubules. These findings suggest that garlic extract reduces oxidative stress and plays a protective role against testicular torsion. Wei et al. [33] observed that testicular I/R in rats led to elevated XO expression, increased ROS production, a significant decline in ipsilateral testicular spermatogenic function, and an increase in MDA content. The impairment of spermatogenic function following I/R was attributed to increased ROS production. There was no significant difference in XO expression in the contralateral testis following allicin administration. However, allicin notably reduced MDA levels and XO expression in the ipsilateral testis [119]. These findings suggest that allicin mitigates testicular I/R injury by suppressing XO expression and reducing ROS production [33] (Figure 4).

### 3.6. Effects of Garlic Derivatives on Skeletal Muscle I/R

I/R injury is a pathological condition that alters the morphology of skeletal muscle tissue, leading to impaired contractile function. Severe skeletal muscle injuries, particularly those accompanied by significant soft tissue damage and rupture of major blood vessels, can result in fatal bleeding. Such injuries are common causes of death in scenarios such as sports fields and battlefields. I/R injury frequently occurs in clinical situations involving skeletal muscles, including limb trauma (especially ischemic muscle damage), severe muscle contusions, and perioperative ischemia during orthopedic surgeries [4]. The wet-weight ratio of muscle is often used as an index to assess muscular edema [120]. Abd et al. [52] developed an I/R model by applying a rubber band around the groin of rats’ hind legs to induce ischemia. After 2 h, the rubber band was removed, allowing reperfusion. An in vitro study examining the I/R wet-to-dry weight (W/D) ratio of the gastrocnemius hind limbs showed that allicin treatment significantly reduced the W/D ratio compared to the control group. These findings indicate that garlic administration effectively decreases IL-1 inflammatory cytokines, increases IL-10 levels, and lowers serum CK expression. Allicin also exhibits anti-apoptotic effects by reducing caspase-3 expression. Overall, these results suggest that garlic can mitigate skeletal muscle I/R-induced injury to varying extents.

## 4. Conclusions and Perspective

This review summarizes the role and recent research progress of garlic and its derivatives in I/R injury. Additionally, the molecular mechanisms through which garlic and its derivatives mitigate I/R-induced injury across multiple organs and tissues, including the heart, brain, kidneys, liver, lungs, and testes, have been thoroughly explained. Garlic and its derivatives alleviate I/R damage by regulating inflammatory factors, oxidative stress, apoptosis, and infarction. Allicin activates the PI3K/Akt/Nrf2 signaling pathway, increases the expression of anti-inflammatory factors IL-4 and IL-10, and reduces the expression of pro-inflammatory cytokines such as IL-6, IL-1, IL-8, ET-1, and TNF-α. Simultaneously, AGE downregulates TNF-α expression, alleviating inflammatory damage caused by ischemia/reperfusion. Allicin alleviates oxidative stress by inhibiting the p-p38 pathway, downregulating MDA, TNF-α, and COX-2 levels, while enhancing the activities of SOD, GPX, and CAT. It also elevates levels of GSH and GST and inhibits ROS-induced Ca^2+^ overload and mitochondrial dysfunction. Other relevant active components exert similar effects: garlic oil reduces ROS, MDA, AST, and ALT and increases CAT levels; DATS lowers MDA and ROS levels, reduces mitochondrial dysfunction, and enhances SOD, GSH, and GPX activities; AGE downregulates COX-2, MDA, AST, and ALT levels, while upregulating SOD, GSH, CAT, and GPX expression; DAS upregulates CAT and SOD levels; and SAC reduces 4-HNE expression.

Allicin also modulates the PI3K/Akt and eNOS/NO pathways, enhancing Bcl-2 expression while suppressing Bax, caspase-3, caspase-9, p-GRK2, p-CaMKII, p-PLC-γ, and p-IP3R levels. This reduces mitochondrial membrane potential, triggers cytochrome C release, and suppresses apoptosis. Other related components similarly regulate apoptosis: DAS increases Bcl-2 expression and decreases caspase-3 expression; DATS increases Bcl-2 expression while decreasing Bax and caspase-3 levels; AGE decreases caspase-3 expression. Allicin also decreases mitochondrial TBARS levels, LDH and CK activities, and Bax expression, while enhancing Bcl-2 expression and elevating HO-1 levels. It reduces LVEF, LVAW, LVDI, and LVID, while enhancing LVD, LVEF, fractional shortening, and stroke volume. Furthermore, other related components also demonstrated similar regulatory effects. Garlic oil reduced the levels of TBARS and HO-1, while DAS was found to downregulate the expression of HO-1. Together, these alterations contribute to reducing infarct size and further underline the protective role of garlic and its derivatives in I/R injury. Garlic and its derivatives hold considerable pharmacological promise for the treatment of I/R injury.

Garlic and its active compounds are widely used as research tools in medical studies and hold significant potential as emerging therapeutic agents for I/R injury in the future [121]. Existing research has shown that garlic and its active substances can act as natural sources of H_2_S precursors, increasing endogenous H_2_S levels [96]. Persulfidation, a core molecular mechanism by which H_2_S regulates protein function, plays a crucial role in its biological effects. This post-translational modification introduces sulfhydryl groups (-SSH) to cysteine residues, thereby altering the conformation, activity, subcellular localization, and interactions of proteins, subsequently modulating cellular signaling pathways, including those involved in oxidative stress, inflammation, and energy metabolism. For instance, persulfidation of p65, a subunit of NF-κB, inhibits its nuclear translocation, thereby blocking the inflammatory response mediated by the NF-κB pathway [122]. Additionally, persulfidation of GAPDH enhances its glycolytic enzyme activity, maintaining cellular metabolic homeostasis [123]. Although this modification has not yet been explored in garlic and its active substances in the context of I/R injury, future studies could identify specific target proteins through mass spectrometry and verify their regulatory effects on related pathways, thereby expanding the understanding of this mechanism. Research on garlic, its derivatives such as polysulfides, and their impact on I/R injury is rapidly gaining momentum. Clinically, allicin injection, a widely used formulation, has been optimized to improve its stability and bioavailability. Currently, allicin injection is used in elderly patients with secondary respiratory infections. Additionally, allicin capsules are used to prevent and treat pulmonary fungal infections in patients with chronic obstructive pulmonary disease, recurrent mouth ulcers, and ulcerative proctitis. These findings demonstrate that allicin preparations can serve as effective antibacterial agents, reduce inflammation, and provide significant therapeutic benefits. Moreover, researchers are exploring innovative formulations such as liposomes and nanoparticles. The continuous development and refinement of allicin-based preparations aim to enhance their efficacy and safety in clinical settings, paving the way for new clinical strategies to treat I/R injuries.

## Figures and Tables

**Table 1:** Garlic and garlic derivatives and ischemia-reperfusion injury. ↓: downregulation, ↑: upregulation.

Action	Allicin (Concentration)	Models	Mechanisms	Publication Date	Reference
**Antioxidant**	Allicin (0.5%)	Myocardial I/R	LDH, CK ↓	2008	Bhatti et al. [27]
	RG (300 mg/kg), ABG (300 mg/kg)	Myocardial I/R	HO-1, iNOS ↑	2018	Czompa et al. [28]
	Allicin (125, 250, 500 mg/kg)	Myocardial I/R	SOD, GSH, and cardiac catalase ↑	2002	Banerjee et al. [29]
	GO (200 mg/kg)	Kidney I/R	TOS, OSI, MPO, NO, and PC ↓	2010	Savas et al. [30]
	Allicin (125, 250, 500 mg/kg)	Myocardial I/R	SOD, CAT, and dp/dt max ↑	2021	Rankovic et al. [31]
	GO (5 mL/kg)	Liver I/R	HO-1, ATG7, and PGC1α ↑	2019	Lasheen et al. [32]
	Allicin (50 mg/kg)	Testicular I/R	xanthine oxidase, ROS ↓	2024	Wei et al. [33]
	Garlic oil (23 mg/kg, 46 mg/kg)	Brain I/R	TBARS ↓	2003	Gupta et al. [34]
	DAS (1.75 mmol/kg)	Liver I/R	HO-1 ↑, CYP2E1 ↓	2008	Shaik et al. [35]
	Allicin (500 mg/kg)	Brain I/R	MDA ↓, GSH, CAT, SOD, Na+-K+ -ATPase ↑	2006	Saleem et al. [36]
	DATS (40 mg/kg)	Brain I/R	Bax, cleaved caspase-3, MDA ↓, AMPK, Bcl-2, GSH, SOD ↑	2023	Sun et al. [37]
	AGE (1 mL/kg)	Liver I/R	AST, ALT, MDA, and GSH ↓	2005	Sener et al. [38]
		Kidney I/R	ROS/MAPK/NF-κARB pathway ↓, SOD, GSH ↑, MDA, ROS ↓	2023	Xu N et al. [39]
**Alleviating mitochondrial dysfunction**	Allicin (1, 10, 50 mg/kg)	Spinal cord I/R	ROS, mitochondrial cytochrome C ↓, CAT, SOD, GPX, GST ↑	2012	Zhu et al. [40]
**Anti-apoptotic**	Allicin (1.2, 1.8, 3.6 mg/kg)	Myocardial I/R	Bcl-2/Bax pathway and LDH, CK ↓	2017	Ma et al. [41]
	Allicin (1.88 mg/kg)	Myocardial I/R	GRK2, PLC-α, IP3R signaling pathways ↓	2021	Gao et al. [42]
	Allicin (0.05%)	Myocardial I/R	Bax/Bcl-2 phosphorylation levels of P-38MAPK and JNK ↓	2012	Sharma et al. [43]
	Allicin (0.2, 1, 5 μM)	H9C2 cell I/H	Bax, MDA ↓, eNOS/NO pathway, Nrf-2, HO-1, Bcl-2, SOD ↑	2018	Ma et al. [44]
	DAS (100, 150, 200 mg/kg)	Brain I/R	caspase-3 ↓, Bcl-2 ↑	2012	Lin et al. [45]
	AGE (250 mg/kg)	Spinal cord I/R	MDA, NO, TNF-α, caspase-3 ↓, SOD, GSH-Px, CAT ↑	2016	Cemil B et al. [46]
		Hypoxia-reoxygenation model of myocardial cells	Bax, cleaved caspase-3, IL-6, TNF-α, cytochrome C ↓, Bcl-2 ↑	2021	Deng X et al. [47]
	Allicin (30 μg/kg)	Lung I/R	P38 MAPK pathway ↑, nuclear factor NF-κAPB, phosphorylated P38 MAPK, Caspase-3, Caspase-9 ↓	2008	Zhang Y et al. [48]
	Allicin (40, 50, 60 mg/kg)	Kidney I/R	Caspase-3, Bax, MDA ↓, Bcl-2 ↑	2022	Li M et al. [49]
**Anti-inflammatory**	Allicin (1.88 mg/kg)	Myocardial I/R	IL-6, TNF-α, EV-1 ↓	2012	Peng et al. [50]
	Allicin (1.2 mL/kg)	Brain I/R	TNF-α, COX-2 ↓	2011	Colín-González et al. [51]
	Allicin (500 mg/kg)	Skeletal muscle I/R	W/D, IL-1 ↓, IL-10 ↑	2019	Abd El-Mottaleb et al. [52]
	Allicin (50 mg/kg)	Myocardial I/R	p38MAPK pathway, p-p38, MDA, TNF-α, IL-6, IL-8 ↓, SOD, CAT, GPx ↑	2019	Liu S et al. [53]
		Liver I/R	IL-1β, TNF-α, PPARγ-IRAK-M-TLR4 signaling pathway ↓	2022	Li W et al. [54]
		Kidney I/R	SOD, IL-4, IL-10, Bcl-2 ↑, MDA, IL-6, TNF-α, Bax, Caspase-3, Cyt-C ↓	2021	Shan Y et al. [55]

Abbreviation: LDH, lactate dehydrogenase; CK, creatine kinase; HO-1, heme oxygenase-1; iNOS, inducible nitric oxide synthase; SOD, superoxide dismutase; GSH, glutathione; TOS, thyroid peroxidase; MPO, myeloperoxidase; NO, nitric oxide; CAT, catalase; dp/dt max, left ventricular pressure derivative; PGC1α, peroxisome proliferator-activated receptor γ coactivator 1α; ROS, reactive oxygen species; TBARS, thiobarbituric acid reactants; CYP2E1, cytochrome P450 2E1; MDA, malondialdehyde; AST, aspartate aminotransferase; ALT, alanine aminotransferase; GST, glutathione S-transferase; GPX, glutathione peroxidase; GRK2, G-protein coupled receptor kinase 2; PLC-α, phospholipase C alpha; Nrf-2, nuclear factor E2-related factor 2; EV-1, Ewing’s sarcoma variant 1; COX-2, cyclooxygenase-2.
